# Pseudomyxoma Peritonei: A Case Report of a Patient With Unexplained Granulomas

**DOI:** 10.7759/cureus.77173

**Published:** 2025-01-09

**Authors:** João Filipe Félix Vieira Afonso, Mafalda Maria Santos, Joana Vieira, Letícia Heeren, Ana Filipa Rodrigues

**Affiliations:** 1 Internal Medicine, Unidade Local de Saúde do Oeste - Caldas da Rainha, Caldas da Rainha, PRT; 2 General Surgery, Unidade Local de Saúde do Oeste - Caldas da Rainha, Caldas da Rainha, PRT

**Keywords:** gastro-intestinal neoplasm, peritoneal pseudomyxoma, peritoneum anomalies, primary infiltrative intestinal type mucinous adenocarcinoma, pseudomyxoma peritonei

## Abstract

Pseudomyxoma peritonei is a rare type of neoplasm, characterized by the presence of mucinous tumors on the peritoneal surface. The authors report the case of a 79-year-old male who came to the hospital with abdominal pain and distension, weight loss, and increased abdominal perimeter. Histopathology is fundamental for making the diagnosis. Survival depends on the extent of the neoplasm. This case reveals the challenges of reaching an accurate and prompt diagnosis of pseudomyxoma peritonei.

## Introduction

Pseudomyxoma peritonei is a rare type of neoplasm. It is characterized by the presence of mucinous tumors on the peritoneal surface, with excessive mucin production [1]. Generally, the primary tumor originates in the mucinous epithelium of the appendix [1,2]. In the latter stages of the disease, it completely involves the peritoneum, leading to symptoms [2,3]. It can also originate from the ovary, pancreas, bile ducts, colon, and gallbladder. Histopathologically, there are four types, which, in ascending order of grading, are acellular mucin, low-grade mucinous carcinoma peritonei or disseminated peritoneal adenomucinosis (DPAM), high-grade mucinous carcinoma peritonei or peritoneal mucinous carcinomatosis (PMCA), and high-grade mucinous carcinoma peritonei with signet ring cells (HMCP-S) or peritoneal mucinous carcinomatosis with signet ring cells (PMCA⁃S) [4]. The estimated incidence is 1:1,000,000-3,200,000 per year, and it is more frequent in females (3:1). The peak incidence is observed in people over 40 years of age [5-7].

Clinically, the spectrum is very variable and nonspecific. 'Jelly belly' is characteristic (30% to 50%), indicating advanced disease progression [3]. Also, at this stage, there is intestinal obstruction, vomiting, nausea, and cachexia, with weight loss. One of the first symptoms is appendicitis-like abdominal pain with intermittent characteristics [1,3]. Symptom recurrence and progression over months or even years are also characteristic [8,9]. The interval between tumor discovery and clinical diagnosis varies greatly [8,10]. Contrast-enhanced CT is the method of choice for the diagnosis, with most cases being diagnosed accidentally. Observation of the primary tumor is very infrequent due to the extent and changes caused by mucin [1,11]. Laparoscopy, for observation and biopsy, seems to be a promising complementary diagnostic tool [12]. The peritoneal cancer index score can be used to evaluate tumor burden during abdominal exploration, confirming the regions in the peritoneum that need to be removed or if an optimal cytoreductive surgery can be performed [13].

Therapeutic approaches are variable and should be patient-focused. For established pseudomyxoma peritonei, a surgical approach is suggested: laparotomy with appendectomy and peritoneal cavity biopsies. Once the diagnosis is confirmed, cytoreductive surgery with the removal of all mucinous tissue, accompanied by adjuvant intraperitoneal radiotherapy/chemotherapy, systemic chemotherapy, or hyperthermic intraperitoneal chemotherapy (HIPEC) [14-18].

## Case presentation

**Figure 1:**
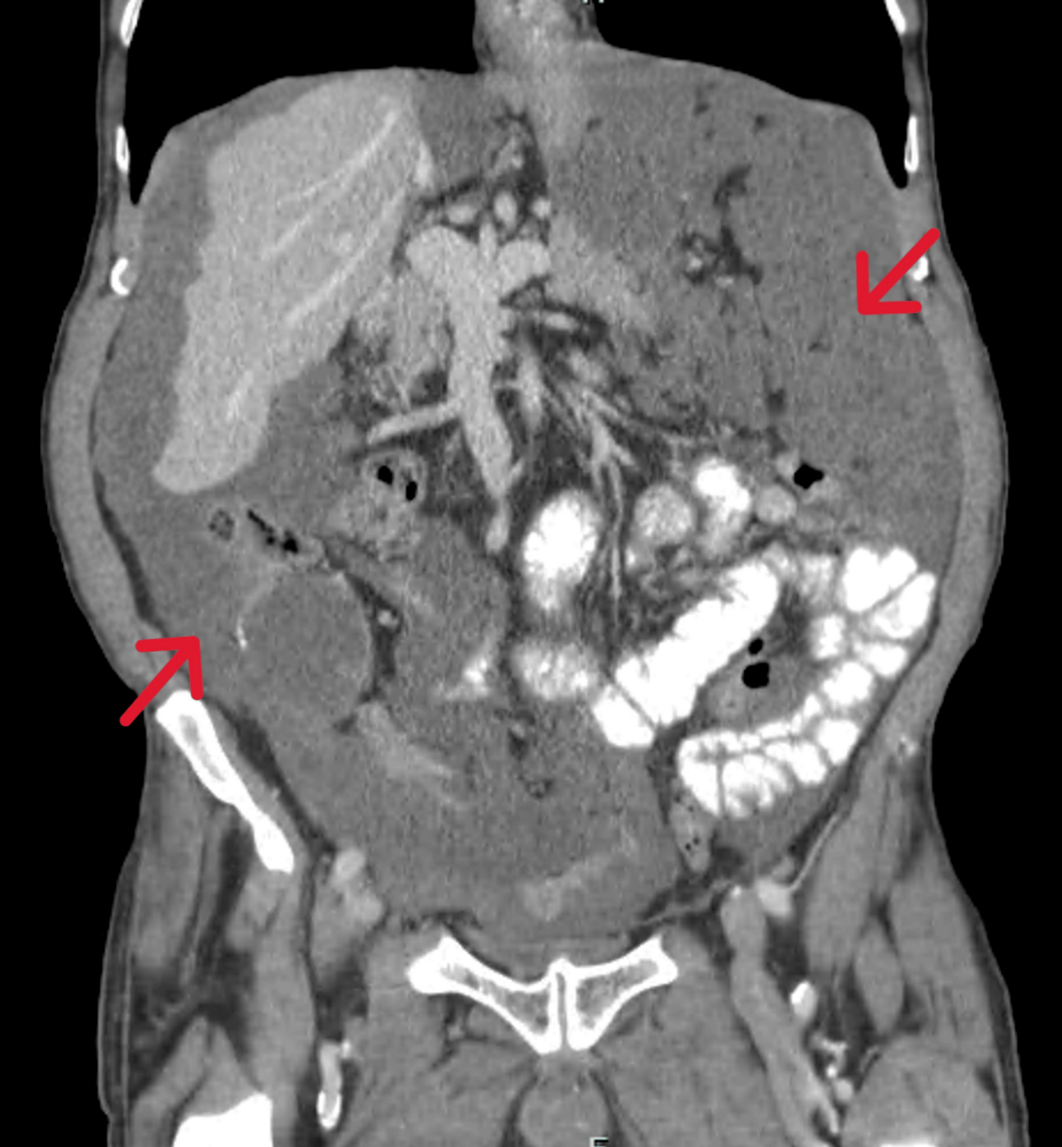
Abdominal CT (coronal view) The red arrows point at a mass compressing all the major intraperitoneal organs.

**Figure 2:**
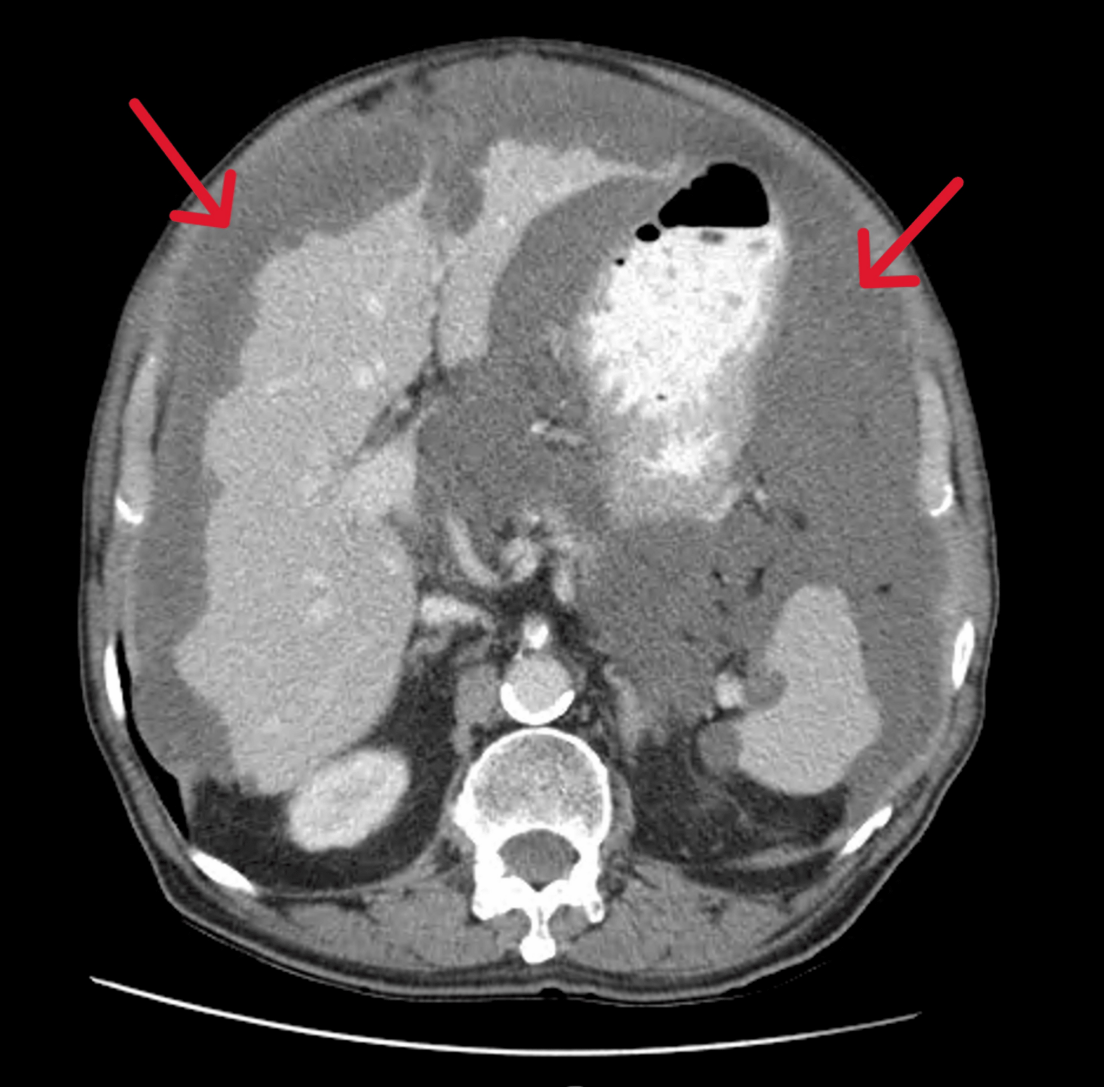
Abdominal CT-Scan (axial view) The red arrows point to the pseudomyxoma's involvement.

A 79-year-old man, with a personal history of ischemic and valvular heart disease and type II diabetes, came to the emergency department with generalized abdominal pain and distension, weight loss of 7 kg, and increased abdominal circumference in the last two months before presentation. He denied fever, night sweats, skin lesions, constipation, diarrhea, and vomiting. He had previously undergone an abdominal CT scan; the scans showed what was described as “granulomas” distributed throughout the peritoneum (Figures 1-2).

On examination, his vitals were blood pressure 123/78 mmHg, heart rate 92 beats per minute, and peripheral oxygen saturation (SpO2) 97% (room air). Notable findings in the physical exam included sarcopenia, collateral abdominal circulation, ascites without tension, and multiple non-tender abdominal nodules. He was hospitalized for further evaluation, considering three main pathologies: neoplasia, granulomatous infection, and granulomatous autoimmune disease.

The patient underwent laboratory tests (Table 1). His antineutrophil cytoplasmic antibodies and angiotensin-converting enzyme were negative. These values remained stable throughout the hospitalization.

**Table 1:** Patient's laboratory parameters CRP: C-reactive protein; AST: Aspartate aminotransferase; ALT: Alanine transaminase; GGT: Gamma-glutamyl transferase; ALP: Alkaline phosphatase; LDH: Lactate dehydrogenase; ESR: Erythrocyte sedimentation rate (ESR); BNP: B-type natriuretic peptide; IgG: Immunoglobulin G; IgM: Immunoglobulin M; IgA: Immunoglobulin A; CEA: Carcinoembryonic antigen; CA 19.9: Cancer antigen 19.9

Parameter	Patient value	Normal range
White blood count	8.1 x 10^3^ u/L	4.0-10.0 X10^3^ u/L
Hemoglobin	10 g/L	13.6-18.0 g/L
CRP	11.1 mg/dL	<0.5 mg/dL
Total bilirubin	0.4 mg/dL	0.2-1.2 mg/dL
AST	14 U/L	5-34 U/L
ALT	18 U/L	0-55 U/L
GGT	10 U/L	12-64 U/L
ALP	64 U/L	40-150 U/L
LDH	123 U/L	125-220 U/L
ESR	120 mm/hour	12-20 mm/hour
BNP	134 pg/mL	<100pg/mL
IgG	1429 mg/dL	540-1822 mg/dL
IgM	45 mg/dL	22-240 mg/dL
IgA	241 mg/dL	101-645 mg/dL
CEA	20.4 ng/mL	<3 ng/mL
CA 19.9	15.9 U/mL	<37 U/mL

**Figure 3:**
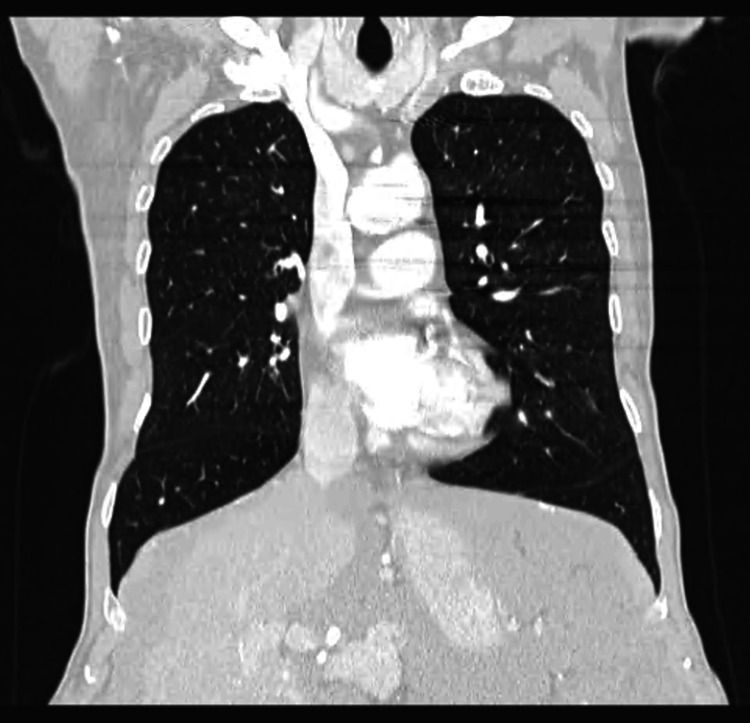
Thoracic CT scan (coronal view). No suspicious lesions were observed.

**Figure 4:**
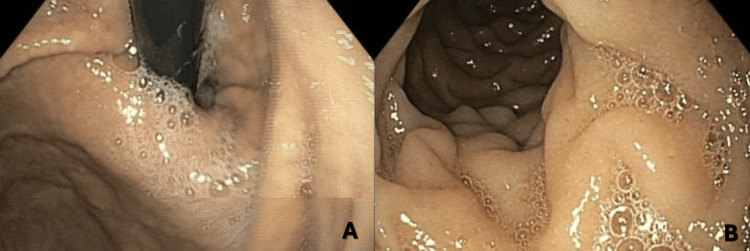
Upper gastrointestinal endoscopy without any signs of neoplasm A: Gastric fundus; B: Duodenum

**Figure 5:**
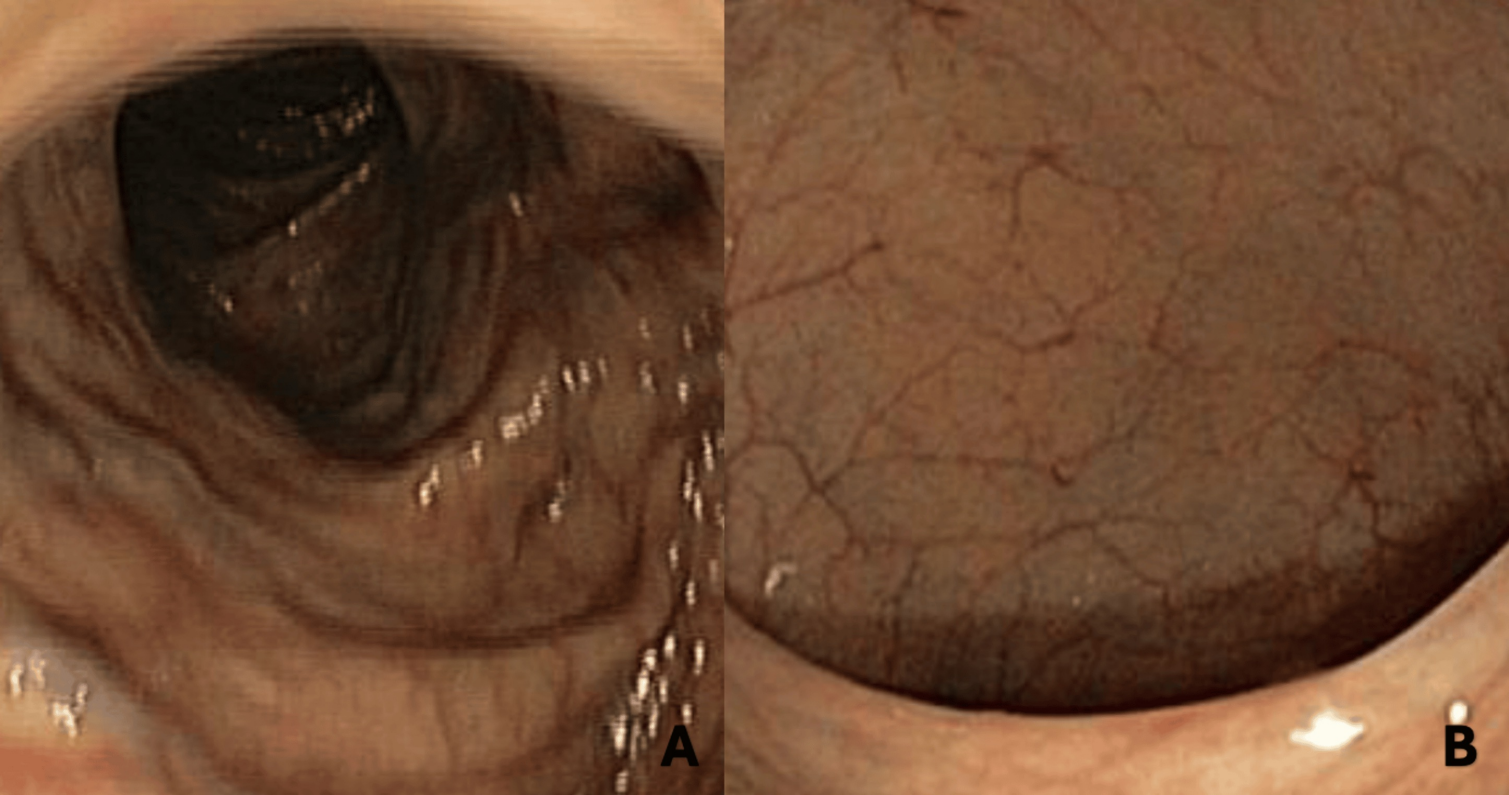
Colonoscopy without obstructions or lesions A: Colon; B: Rectum

The patient's HIV, interferon-gamma release assay, and hepatitis tests were negative. A positive treponemal test (titer dilution 1:1280) indicated untreated late syphilis, so benzylpenicillin was started. Simultaneously, further diagnostic workup included thoracic CT (Figure 3), upper gastrointestinal endoscopy (Figure 4), and colonoscopy (Figure 5), all without suggestive aspects of obstruction and neoplasm. An abdominal MRI was performed, showing signs of peritoneal carcinomatosis suggestive of intestinal neoplasm, but the origin could not be identified.

**Figure 6:**
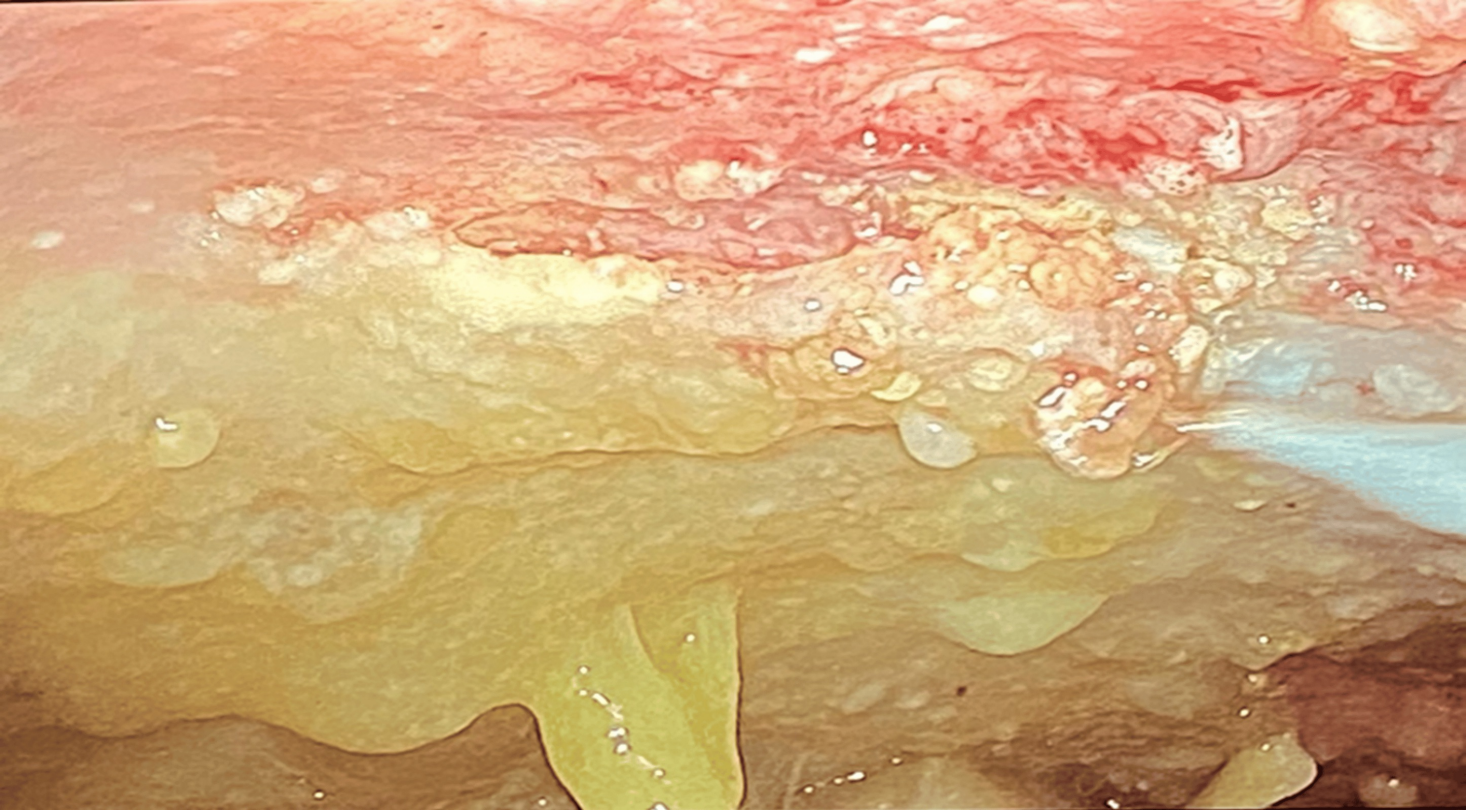
Abdominal laparoscopic images revealing the peritoneum wall with the mucinous tumour

After discussing the case with General Surgery, the patient was proposed for exploratory laparoscopy with excisional biopsy of the peritoneal lesions (Figure 6). Histology revealed a mucinous adenocarcinoma of the peritoneum. The patient was referred to a peritoneal tumor unit at a reference hospital as soon as the diagnosis was made, where he started palliative intraperitoneal chemotherapy.

## Discussion

In pseudomyxoma peritonei, the primary tumor usually originates in the mucinous epithelium of the appendix [1,2]. As the disease progresses, it completely involves the peritoneum, leading to symptoms such as abdominal pain, anorexia, and cachexia, as our patient showed [1,3]. Contrast-enhanced CT is the gold-standard method for diagnosis. However, identification of the primary tumor is very difficult [1,11]. In our extended investigation, we could not find the primary tumor.

One of the possible differential diagnoses was granulomatous infections. Our patient had late syphilis, which could have been a confounder (with gummatous syphilis), hence the importance of histological diagnosis. As for granulomatous vasculitis and IgG4 disease, the antibodies were negative, as the IgG levels were within the normal range. No other symptoms suggestive of these autoimmune diseases were present. Only histopathology could rule out these differential diagnoses that occurred in our case.

Cytoreductive surgery with adjuvant intraperitoneal radiotherapy/chemotherapy or systemic chemotherapy or HIPEC is the current therapeutic approach [14-18]. Our patient, due to his comorbidities, was not a candidate for curative treatment, so he was proposed only for palliative intraperitoneal chemotherapy. Pseudomyxoma peritonei presents a slowly progressive behavior, with survival reaching up to 20 years. Five- and ten-year survival rates after conventional cytoreductive surgery range from 53% to 75% and 32% to 60%, respectively, with a median survival from diagnosis of 75 months [1,3,18,19]. The main predictors of poor overall survival include older age, major postoperative complications, completeness of cytoreduction, prior chemotherapy treatment, and PMCA [20].

## Conclusions

Pseudomyxoma peritonei is a rare neoplasm that is poorly understood. Its diagnosis is based on a combination of clinical presentation, characterized by mucinous ascites and abdominal distension, and a CT scan. Though treatment approaches are not yet standardized, combining peritonectomy and HIPEC appears to improve prognosis. Patients with high-grade mucinous adenocarcinoma of the peritoneum involving five abdominal regions and/or intestinal involvement should undergo palliative treatment.
